# Hematologic Tumor Cell Resistance to the BCL-2 Inhibitor Venetoclax: A Product of Its Microenvironment?

**DOI:** 10.3389/fonc.2018.00458

**Published:** 2018-10-22

**Authors:** Joel D. Leverson, Dan Cojocari

**Affiliations:** ^1^Oncology Development, AbbVie, Inc., North Chicago, IL, United States; ^2^Oncology Discovery, AbbVie, Inc., North Chicago, IL, United States

**Keywords:** venetoclax, BCL-2, microenvironment, resistance, tumor

## Abstract

BCL-2 family proteins regulate the intrinsic pathway of programmed cell death (apoptosis) and play a key role in the development and health of multicellular organisms. The dynamics of these proteins' expression and interactions determine the survival of all cells in an organism, whether the healthy cells of a fully competent immune system or the diseased cells of an individual with cancer. Anti-apoptotic proteins like BCL-2, BCL-X_L_, and MCL-1 are well-known for maintaining tumor cell survival and are therefore attractive drug targets. The BCL-2-selective inhibitor venetoclax has been approved for use in chronic lymphocytic leukemia and is now being studied in a number of other hematologic malignancies. As clinical data mature, hypotheses have begun to emerge regarding potential mechanisms of venetoclax resistance. Here, we review accumulating evidence that lymphoid microenvironments play a key role in determining hematologic tumor cell sensitivity to venetoclax.

## Introduction

### The BCL-2 family: arbiters of cell survival and programmed cell death

**Figure 1:**
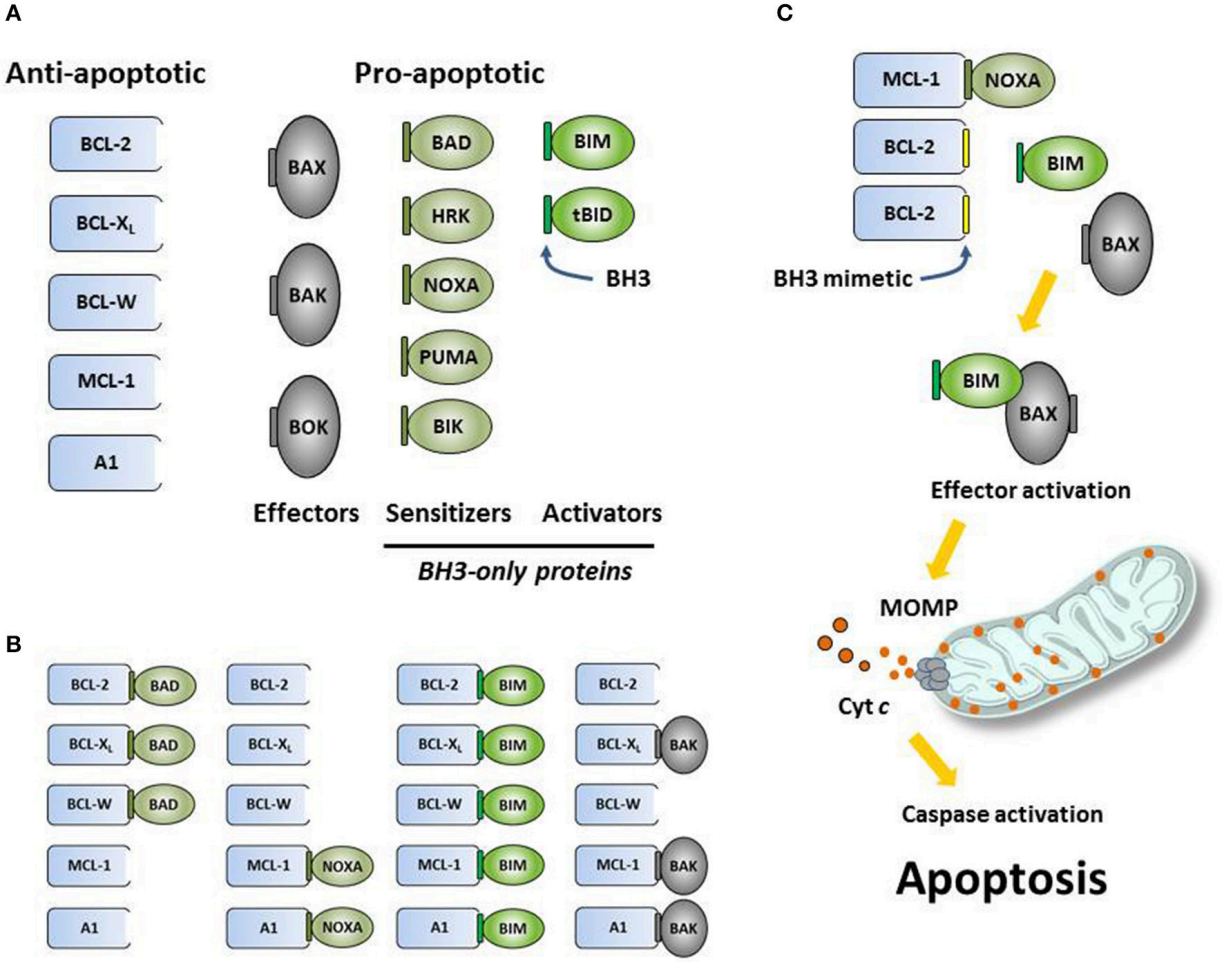
**(A)** The intrinsic (mitochondrial) pathway of apoptosis is regulated by structurally related proteins in the BCL-2 family, which share from one to four BCL-2 homology (BH1-BH4) motifs. These proteins can be sub-classified as anti-apoptotic (pro-survival) or pro-apoptotic (pro-death). Pro-apoptotic proteins can be further sub-divided into multi-BH effector proteins (BAX, BAK, BOK) and so-called BH3-only proteins. Certain BH3-only proteins like BIM can bind and allosterically activate effector proteins, promoting their insertion into mitochondrial membranes and subsequent oligomerization. Other BH3-only proteins, such as NOXA, can act as sensitizers of apoptosis by binding to anti-apoptotic proteins and precluding their sequestration of pro-apoptotic effectors and activators. **(B)** Anti-apoptotic proteins bind the BH3 motifs (depicted as small, green rectangles) of specific pro-apoptotic proteins, thereby sequestering them and preventing the initiation of apoptosis. Each pro-apoptotic protein demonstrates its own selectivity profile regarding which anti-apoptotic protein(s) it tends to associate with. **(C)** Synthetic small-molecule “BH3 mimetics” (depicted as small, yellow rectangles) like venetoclax are designed to bind certain anti-apoptotic proteins and compete for binding with pro-apoptotic proteins. Pro-apoptotic proteins liberated by BH3 mimetics are free to initiate the key molecular events of programmed cell death, including effector activation, mitochondrial outer membrane permeabilization (MOMP), the release of apoptogenic factors like cytochrome *c* (depicted as small red circles) into the cytosol, the proteolytic activation of caspases and the dismantling of the cell.

The *BCL2* gene was discovered as part of the *t*(14;18) translocation associated with follicular lymphoma (1) and was later characterized as the first oncogene to work by maintaining tumor cell survival (2–5). Scientists went on to discover a host of related proteins that now comprise the BCL-2 family (Figure 1A) [see (6) for review]. These proteins are characterized by closely related structural units known as BCL-2 homology (BH) motifs—a collection of alpha-helices that assemble to form surfaces that mediate interactions amongst family members. The BH1-BH4 motifs of anti-apoptotic proteins such as BCL-2, BCL-X_L_ and MCL-1 form a shallow, hydrophobic groove that accommodates binding of the amphipathic BH3 motif of certain pro-apoptotic family members like the multi-domain “effector” proteins BAK and BAX. Each BCL-2 family member exhibits a binding selectivity profile reflecting its tendencies to interact more avidly with certain counterparts (Figure 1B). For example, the effector protein BAK tends to be sequestered by BCL-X_L_, MCL-1, or A1, whereas BAX exhibits binding to all the anti-apoptotic proteins. Likewise, all anti-apoptotic proteins are thought to be capable of sequestering the so-called “BH3-only” protein BIM, a pro-apoptotic “activator” that can promote the insertion of BAX into the mitochondrial outer membrane. Thus activated, BAX can oligomerize and form complexes with BAK to form pores in the mitochondrial outer membrane (Figure 1C). When so-called “sensitizer” proteins bind to anti-apoptotic counterparts, they can preclude sequestration of activators and effectors, thereby promoting apoptosis. For example, the pro-apoptotic protein BAD binds to BCL-2, BCL-X_L_, and BCL-W but not to MCL-1, whereas NOXA binds preferentially to MCL-1 and A1 (Figure 1B). Certain cellular stresses can lead to elevations in pro-apoptotic proteins, which can then overwhelm the anti-apoptotic proteins and go on to trigger the key events of intrinsic apoptosis, including mitochondrial outer membrane permeabilization (MOMP) by BAK-BAX oligomers, the release of mitochondrial cytochrome *c* into the cytosol, the proteolytic activation of caspases, and the eventual dismantling of the cell and its engulfment by macrophages (Figure 1C).

For cancer cells, which often must evolve to survive in harsh environments, the overexpression of anti-apoptotic proteins allows increased numbers of pro-apoptotic proteins to be sequestered, offering a mechanism of survival, and a selective advantage. However, because they carry such high levels of these complexes, these cells essentially exist on the brink of initiating apoptosis, a state which has been referred to as “primed for death” (7). In an attempt to exploit this therapeutically, small-molecule BH3 mimetics have been designed to bind competitively to anti-apoptotic proteins and liberate pro-apoptotic proteins in the hopes of triggering apoptosis in primed cancer cells (Figure 1C) [see (8) for review]. Decades of intense drug discovery efforts have recently borne fruit with regulatory agency approvals of venetoclax, a selective BCL-2 inhibitor.

### The BCL-2-selective inhibitor venetoclax

The first BH3 mimetics, such as ABT-737 and ABT-263 (navitoclax), exhibited the same binding profile as the BH3-only protein BAD, inhibiting BCL-2, BCL-X_L_, and BCL-W (9, 10). This profile accounted for both the early anti-tumor activity that was observed in CLL (11) and the dose-limiting toxicity of thrombocytopenia, with BCL-2 inhibition driving the former and BCL-X_L_ inhibition the latter (12, 13). Based on these findings, drug discovery scientists designed BCL-2-selective agents, such as ABT-199/venetoclax and S55746/BCL201, which maintain killing activity against CLL cells while sparing platelets (8, 14). Venetoclax was the first BCL-2-selective agent to enter the clinic and quickly showed signs of anti-tumor activity. Tumor lysis syndrome (TLS) was observed in two of the first three CLL patients to receive a dose (14) and objective response rates nearing 80% were reported for relapsed/refractory patients, including those with high-risk forms of the disease (15). Based on these and other data, venetoclax was approved by the FDA for use in relapsed/refractory CLL with 17p deletion. A host of other clinical trials are now under way, including combination studies in CLL, acute lymphocytic leukemias, myeloid leukemias, non-Hodgkin lymphomas, and breast cancer [see (16) for review].

## Predicting mechanisms of resistance to venetoclax

As the first encouraging signs of venetoclax activity were being observed in the clinic, translational scientists were already at work, hoping to anticipate mechanisms of resistance that might emerge. Early efforts focused on cancer cell lines that acquired resistance after prolonged culture with venetoclax. By comparing the parental cells to the resistant populations that emerged, a variety of potential resistance mechanisms were identified. Unlike the very specific “gatekeeper” mutations that primarily account for tyrosine kinase inhibitor resistance in CML, a more diverse array of alterations were observed in the cell lines exhibiting venetoclax resistance. Not surprisingly, resistance in some cell lines was associated with elevations in anti-apoptotic proteins such as BCL-X_L_ or MCL-1 (17), which can serve to back up BCL-2. Conversely, pro-apoptotic proteins like BIM and BAX were seen to be mutated, reduced or even lost in resistant populations (17, 18). There were also some surprising cases, analogous to gatekeeper mutations, in which BH3-binding pocket mutations in BCL-2 reduced venetoclax binding while apparently retaining affinity for endogenous pro-apoptotic ligands. Mutations in phenylalanine 101 (F101C, F101L) of murine Bcl-2 were identified in venetoclax-resistant murine cell lines (18) while, in a separate lab, the corresponding mutation (F103) was observed in a resistant population of the human cancer cell line SC-1 (17). Taken as a whole, these findings indicate that numerous, distinct mechanisms could account for resistance to venetoclax when given as monotherapy.

## Venetoclax resistance and the tumor microenvironment

While these first clues about cancer cell-intrinsic mechanisms of venetoclax resistance were emerging, other labs began to explore the role of extrinsic factors found in the tumor microenvironment. Like normal hematopoietic cells, which rely on interactions with stromal cells and certain immune cells as they develop and differentiate, cancer cells retain a dependence on supportive cells in lymphoid organs such as the bone marrow, spleen and lymph nodes. Within these organs stromal cells and immune cells deposit extracellular matrix and secrete growth factors, chemokines, and interleukins that provide tumor cells with homing, adhesion, growth, proliferation and survival signals [see (19) for an excellent review]. For example, malignant B-cells receive survival signals from supporting T follicular helper (T_FH_) cells expressing the CD40 ligand (CD40L), which drives NFκB signaling downstream of CD40 engagement. B-cell receptor (BCR) signaling, crucial to normal B-cell survival and development, also remains active in most lymphomas and certain leukemias, either as a function of self-antigen engagement in the tumor microenvironment or through mechanisms that leave the BCR constitutively activated and antigen-independent. Toll-like receptors (TLR) like TLR9 have also shown a role in mediating tumor cell survival signals originating in lymphoid organs.

## Venetoclax resistance in chronic lymphocytic leukemia

Researchers exploring these concepts and their potential impact on venetoclax resistance began to recognize some familiar themes. Just as previous work had demonstrated that kinase signaling cascades downstream of CD40 engagement signal the upregulation of anti-apoptotic proteins like BCL-X_L_, MCL-1 and BFL-1/A1 in B-cells (20–24), so CLL cells co-cultured with CD40L-expressing fibroblasts were found to upregulate BCL-X_L_, MCL-1 and BFL-1 (25)—changes that rendered these cells essentially insensitive to venetoclax. Consistent with other reports (26), BCL-X_L_ seemed to play the most prominent role in this resistance, as its siRNA-mediated silencing, but not that of MCL-1, led to some re-sensitization of these cells to venetoclax. Based on the elucidation of signaling pathways known to function downstream of CD40, these teams began to assess kinase inhibitors that might resensitize tumor cells to venetoclax. ABL tyrosine kinase inhibitors like imatinib and dasatinib were able to prevent CD40L-dependent upregulation of BCL-X_L_, MCL-1, and BFL-1 and reverse resistance to venetoclax, whereas BCR signaling inhibitors like the BTK inhibitor ibrutinib and the PI3Kδ inhibitor idelalisib had little effect. Similarly, in another study of venetoclax resistance mediated by BCR pathway stimulation, ibrutinib and idelalisib were less effective than the SYK tyrosine kinase inhibitors R406 and entospletinib at reducing MCL-1 levels and sensitizing CLL cells to venetoclax (27). The SYK/JAK inhibitor cerdulatinib has also been shown to synergize with venetoclax by inhibiting the upregulation of BCL-X_L_ and MCL-1 in CLL cells treated with CD40L and IL-4 or co-cultured with nurse-like cells (28). Significant resistance to the BCL-2/BCL-X_L_ inhibitor ABT-737 was also observed in CLL cells cultured in the presence of IL-4 and CD115-expressing fibroblasts, which induced the expression of BCL-X_L_ and BCL2A1 (22). A phase 1 study is currently under way to explore the combination of venetoclax and the SYK inhibitor TAK-659 for patients with relapsed/refractory NHL (NCT03357627). The cytoplasmic tyrosine kinase LYN has also been implicated as a mediator of microenvironment-mediated CLL cell survival (29) and may play crucial roles in the supporting stromal cells themselves.

Although these studies suggested that BTK inhibitors and PI3K inhibitors may not be ideally suited for counteracting venetoclax resistance when tumor cells are residing in protective niches, it is important to note that these inhibitors are highly effective at mobilizing tumor cells out of those niches into peripheral circulation. In fact, it is common to observe large elevations in circulating lymphocytes (lymphocytosis) in the first 1–2 months of ibrutinib treatment, as abnormal B-cells migrate out of lymphoid organs upon disruption of BCR signaling (30). Based on the co-culture experiments described above, the prediction would be that these cells should be particularly susceptible to venetoclax-mediated killing while in circulation. Indeed, residual tumor cells isolated from the blood of CLL patients taking BTK inhibitors such as ibrutinib or acalabrutinib have been shown to be highly sensitive to venetoclax (31, 32). Similar results were observed when venetoclax was added to mantle cell lymphoma (MCL) cells isolated from circulation after ibrutinib treatment (33). Moreover, early data from clinical studies exploring the combination of venetoclax and ibrutinib have shown impressive objective response rates, including high rates of minimal residual disease (MRD)-negativity (see section below).

Other kinase signaling pathways have also been implicated in stroma-mediated venetoclax resistance. CLL cells collected from peripheral blood were shown to upregulate MCL-1 when co-cultured with NK-tert bone marrow stromal cells (34). Although cyto-protective, the interaction with stromal cells did not induce proliferation of the CLL cells. Stroma-mediated elevations in MCL-1 were associated with increased AKT and MAPK/ERK signaling, which may reduce MCL-1 proteolysis, as well as increased phosphorylation of serine 5 of the RNA polymerase-II C-terminal domain, which is mediated by CDK9 and known to support the elongation of *MCL1* transcripts. Other studies support the idea that combinations with MEK (35) or CDK9 (36–38) inhibitors could enhance venetoclax activity and circumvent resistance, and ongoing clinical studies in acute myeloid leukemia (AML) may soon provide clinical data (see below).

While most early resistance studies focused specifically on alterations in BCL-2 family members (a rational starting point), more recent work has begun to explore venetoclax resistance in an unbiased fashion. For example, Herling et al. performed whole-exome sequencing of samples from CLL patients before receiving venetoclax and after developing resistance (39). Similar to the work done *in vitro*, these studies identified a number of potential resistance-associated alterations, including mutations in *BTG1* or *BRAF*, homozygous deletion of *CDKN2A/B* and high-level focal amplification of *CD274*, the gene encoding the immune checkpoint protein PD-L1. Although the sample size of this study was small (*n* = 8) and the causative role of these potential resistance mutations remain to be confirmed, it is anticipated that data accrued from this and similar unbiased analyses will continue to define novel venetoclax resistance mechanisms.

## Venetoclax resistance in non-hodgkin lymphomas

Although the early results from venetoclax studies in CLL were highly encouraging, data from studies in follicular lymphoma (FL) and diffuse large B-cell lymphomas (DLBCL) have been less compelling. In a monotherapy study, objective response rates of 38 and 18% were reported for FL and DLBCL, respectively (40). These results were somewhat perplexing, given the fact that these tumors are often defined by the *t*(14;18) translocation, which drives high-level expression of BCL-2 in most cases. Although preclinical studies using FL and DLBCL cell lines had suggested a strong correlation between *t*(14;18)-positivity or *BCL2* gene amplification and sensitivity to venetoclax *in vitro* (14), the link does not seem as strong in the clinic. While disappointing, this may not be surprising given the potential intratumoral heterogeneity of BCL-2 expression in follicular lymphomas (41). One possibility is that the *t*(14;18) translocation is a crucial driver of tumor initiation but, as the cancer evolves, becomes dispensable for survival and tumor maintenance.

In a recent study, *t*(14;18)-positive lymphoma cells were treated with venetoclax for an extended period to induce resistance (42). Comparing the venetoclax-resistant and parental cell lines, the resistant FL cells had significantly higher levels of ERK1/2 and BIM phosphorylation at serine 69. Phosphorylation of BIM at serine 69 has been shown to target BIM for proteasomal degradation, thus reducing the pro-apoptotic priming of the cells (43). Targeting the cell surface protein CD20 with the chimeric monoclonal antibody rituximab prevented the phosphorylation of ERK1/2 and BIM, and improved the activity of venetoclax in xenograft models of these FL cells (42). Similar findings were reported in MCL (44).

The influence of the tumor microenvironment in lymphomas (19) could also account for the weaker-than-expected efficacy signals. FL cells are known to split time between peripheral circulation and germinal centers, where processes like activation-induced cytidine deaminase (AID)-mediated mutagenesis could drive clonal evolution and acquired dependencies on other anti-apoptotic proteins (45). Similarly, lymphoma cells may simply upregulate other BCL-2 family survival proteins while residing in lymph nodes, making them distinct from cell lines that are cultured in monolayers *in vitro*. Indeed, MCL cells co-cultured with CD40L-expressing fibroblasts were shown to express elevated levels of BCL-X_L_ downstream of NFκB signaling (33, 44). Jayappa et al. described a similar mechanism in response to CD40, IL-10 or TLR9 agonists that can account for the resistance of MCL cells to venetoclax-ibrutinib combinations (46).

## Venetoclax resistance in myeloid malignancies and multiple myeloma

Although most of its early clinical trials were focused on B-cell malignancies like CLL and NHLs, venetoclax has also begun to show activity in myeloid malignancies. For example, a Phase 2 study exploring venetoclax as monotherapy in patients with relapsed/refractory AML reported a 19% objective response rate (47), though the durability of responses was limited. Sequencing of paired patient samples from that study indicated that FMS-like tyrosine kinase (FLT3) mutations are associated with basal and acquired resistance (48), and so combinations with FLT3 inhibitors like quizartinib or gilteritinib would therefore represent rational combinations. Venetoclax combinations with standard-of-care agents such as hympomethylating agents (HMAs) and low-dose cytarabine (LoDAC) are already being explored in elderly, treatment-naïve populations who are unfit for high-intensity induction regimens. Objective response rates (ORR), which include complete responses (CR), complete responses with incomplete bone marrow recovery (CRi) and partial responses (PR), have been reported as 62% for combination with LoDAC (49) and 61–67% for combinations with HMAs (50, 51)—well above historical values reported for those agents on their own. Based on these data, the FDA granted breakthrough therapy designation for both combinations. Venetoclax combinations with CDK9 inhibitors like alvocidib (NCT03441555) or dinaciclib (NCT03484520) are also being pursued, with the hypothesis that these agents will synergize with venetoclax based on their ability to inhibit MCL-1 expression [see (36–38)]. There is also optimism that BH3 mimetics such as AMG176, which can inhibit MCL-1 directly [see (8) for review], will prove safe enough in ongoing AML and multiple myeloma studies (NCT02675452) to combine with venetoclax.

Plasma cells are known to depend on MCL-1 for survival and, following malignant transformation, multiple myeloma cells appear to preserve this dependency. However, studies using cell lines or *ex vivo* cultures of patient cells treated with venetoclax or ABT-737 have shown that there are also subsets of myeloma cells that are primarily BCL-2-dependent (52, 53). A Phase 1 trial of venetoclax monotherapy showed that myeloma patients with *t*(11;14)-positive tumors, which tend to express high levels of BCL-2 and low levels of BCL-X_L_ and MCL-1, showed an objective response rate of 40% (54). In the non-*t*(11;14) population, BCL-X_L_ and/or MCL-1 are likely to play a larger role in maintaining myeloma cell survival, and the tumor microenvironment likely plays a role in driving their expression. Some studies have indicated that bone marrow stromal cell-derived cytokine Interleukin-6 (IL-6) can upregulate MCL-1 and BCL-X_L_ expression in myeloma cells, thus providing a possible mechanism of resistance to venetoclax (55). More recent studies have revealed that IL-6 may also influence sensitivity to venetoclax through mechanisms other than regulating the expression of BCL-2 family proteins (56). Using an immortalized bone marrow stromal cell line or conditioned media from these cells, the authors induced resistance to either venetoclax or ABT-737 in myeloma cell lines, and this resistance was reversed by a neutralizing IL-6 antibody. Interestingly, IL-6 did not alter the expression of BCL-2 family member proteins but instead shifted BIM binding from BCL-2/BCL-X_L_ to MCL-1. This shift in priming occurred through the ERK1/2-mediated phosphorylation of serines 69 and 77 on BIM, similar to an acquired resistance mechanism observed with venetoclax in FL (42). As a result, the shift to MCL-1:BIM priming, and thus MCL-1 dependence, was prevented by inhibitors of either JAK1/2 or MEK signaling pathways.

Despite the observed synergy between JAK and BCL-2/BCL-X_L_ inhibition in myeloma it was unclear whether the tumor microenvironment plays a similar role in other malignancies, and whether JAK inhibitors might combine with venetoclax to counteract bone marrow stroma-mediated resistance in those diseases. One team screening a panel of 304 inhibitors against AML patient samples identified bone marrow stromal cell conditions that significantly reduced responses to around 10% of the molecules (57). In the presence of cytokines from stromal cell-conditioned media, AML cell killing mediated by venetoclax was significantly lower. The cytokines activated JAK/STAT signaling to support AML cell proliferation and survival and decreased the expression of BCL-2 relative to BCL-X_L_. Unlike multiple myeloma, where IL-6 was found to be crucial, GM-CSF was the essential stroma-derived factor for AML cell survival. The JAK2 inhibitor ruxolitinib was more active in the presence of the cytokine-rich media and, when combined with venetoclax, demonstrated synergistic killing activity. This result was recapitulated in a systemic xenograft model of AML. Another team employing an *ex vivo* drug sensitivity profiling screen using freshly isolated patient samples identified the venetoclax-ruxolitinib combination as the most active in killing malignant myeloid cells (58). Despite the lack of stromal cell culture media in these screens, drug sensitivity was evaluated *ex vivo* within 24 h of sample collection, which may have preserved the bone marrow stromal effects.

Most of the tumor cell resistance mechanisms described here have focused on the modulation of BCL-2 family proteins that can occur downstream of stromal cell engagement. However, the interplay between tumor cells and cells in their microenvironment may be even more complex. Intriguing new work has begun to show that metabolites and organelles, including some as large as mitochondria, can be transferred between cells, including cancer cells and their “normal” neighbors in tumor microenvironments. One study recently described how AML cells, which are thought to be reliant on oxidative phosphorylation (OxPhos) to generate energy, can (mis)appropriate the mitochondria of stromal cells in the bone marrow, with the apparent survival benefit of enhanced OxPhos capacity. In an elegant series of experiments, Marlein et al. showed that AML cells can accomplish this mitochondrial pilfering through the use of tunneling nanotubes (TNTs), filamentous actin-based structures that may exceed 200 nm in diameter (59). In order to visualize this process, the plasma membranes of AML cells were labeled with a red dye to distinguish them from co-cultured bone marrow stromal cells. The latter were labeled with green Mito tracker, making it possible to track the localization and any inter-cellular migrations of stromal cell mitochondria. Intriguingly, red-labeled TNTs could be observed extending out from AML cells to contact neighboring stromal cells. In addition, speckles of red dye could be observed pock-marking the surface of stromal cells that had thus been probed. These TNT access points, or “TAPs,” seemed to be concentrated on specific stromal cells, which the investigators took as a clue that some form of active signaling might be involved. The group went on to show that NADPH oxidase-2 (NOX2) on the surface of AML cells may produce concentrated zones of superoxide, which stromal cells read as a signal to increase production of mitochondria. Indeed, proliferator-activated receptor gamma coactivator 1-alpha (PGC-1α) signaling was found to be upregulated in the stromal cells, and drove the increased expression of genes encoding mitochondrial components (60). Once this crop of mitochondria has been produced, the AML cells begin TAPping these cells to harvest the mitochondria and reap the benefit of their enhanced OxPhos capacity.

It is tempting to speculate that AML cells thus acquiring “foreign” mitochondria, could acquire a new BCL-2 family dependence profile based on the complement of anti-apoptotic proteins populating those mitochondria. While these mitochondrial profiles would not be inherited permanently (BCL-2 family proteins are not encoded by mitochondrial genes), it is conceivable that such a mechanism could provide enough survival advantage to promote the outgrowth of certain sub-clones. It will be interesting to see how this nascent field matures and whether therapeutic strategies to target these microenvironment-driven mechanisms prove effective.

## Overcoming tumor microenvironment-mediated venetoclax resistance

Based on the mechanisms of venetoclax resistance that have been observed preclinically, a number of rational combination hypotheses have emerged. For example, CD20 antibodies such as rituximab and obinutuzumab were shown to reverse venetoclax resistance that occurred when CLL cells were co-cultured with stromal cells (25). Because these agents are standards-of-care for many B-cell malignancies, their combination with venetoclax was already being explored clinically. Strong activity in a single-arm Phase 1b study of relapsed/refractory CLL combining venetoclax with rituximab (ORR: 86%, CR: 51%, MRD-negativity in bone marrow: 57%; *n* = 49) (61) led to the granting of breakthrough therapy designation by the FDA, and data were recently reported for the randomized Phase 3 study MURANO (62), which compared venetoclax-rituximab to the combination of rituxumab and the alkylating agent bendamustine in patients with relapsed/refractory CLL. That study reported an ORR of 93.3% and a CR/CRi rate of 26.8% for the venetoclax-rituximab arm, per investigator assessments (ORR: 92.3%, CR/CRi: 8.2% by independent review committee). The median progression-free survival (mPFS) of patients receiving venetoclax-rituximab (*n* = 194) had not been reached after a median follow-up of 24.8 months, compared to a median PFS (investigator-assessed) of 17 months for the bendamustine-rituximab arm (*n* = 195) (hazard ratio: 0.17, 95% confidence interval: 0.11–0.25, *p* = 0.0001). Independent review committee assessments were similar, with mPFS for the venetoclax-rituximab arm not reached, vs. 18.1 months for patients receiving bendamustine-rituximab (hazard ratio: 0.19, 95% confidence interval: 0.13–0.28, *p* = 0.0001). These data led the FDA to grant full approval of venetoclax in combination with rituximab for patients with CLL having received at least one prior therapy. A Phase 1b study of venetoclax plus obinutuzumab in previously untreated CLL reported an ORR of 100% and a CR/CRi rate of 72% (*n* = 32) (63). Similarly, the CLL14 Phase 3 study (venetoclax plus obinutuzumab in previously untreated CLL patients with coexisting medical conditions) reported an ORR of 100% and a CR rate of 58%, with MRD-negativity in peripheral blood of 92% (*n* = 12) (64).

Based on their ability to mobilize leukemia cells out of protective lymphoid niches, BCR pathway inhibitors are also being explored in combination with venetoclax. An initial period of tumor debulking is typically implemented with the mobilizing agent alone to mitigate the risk of tumor lysis syndrome associated with venetoclax. Results from ongoing studies of venetoclax and ibrutinib have been particularly promising. In the CLARITY study, an objective response rate of 100% was reported, with 60% CR/CRi (*n* = 25) (65). When assessed in bone marrow, an MRD-negativity rate of 28% was observed. A separate Phase 2 study of venetoclax combined with ibrutinib includes a cohort of treatment-naïve CLL patients and has reported an overall response rate of 100%, with CR/CRi and MRD-negativity rates increasing over time (CR/CRi: 61%, MRD-negativity: 21% after 3 months of combination, *n* = 33; CR/CRi: 75%, MRD-negativity: 45% after 6 months of combination, *n* = 20; CR/CRi: 80%, MRD-negativity: 80% after 9 months of combination, *n* = 10) (66). There are also preclinical data demonstrating synergy between venetoclax and PI3K inhibitors like the PI3Kδ inhibitor idelalisib and the dual PI3Kδ/PI3Kγ inhibitor duvelisib (67). SYK inhibitors like entospletinib (27) or cerdulatinib (28) have also shown promise preclinically. The cytoplasmic tyrosine kinase LYN may also be a good target, having roles in BCR signaling as well as in cells of the tumor microenvironment (29).

## Conclusion and future directions

The studies described here are excellent examples of how preclinical data can inform improved clinical strategies. With the identification of potential mechanisms of venetoclax resistance mechanisms have come clear hypotheses for combination strategies to avert or reverse it. Some of these hypotheses are already being tested clinically and are showing signs of promise. Venetoclax combinations with CD20 antibodies or BCR pathway inhibitors have shown clear improvements in efficacy relative to the respective monotherapies. Moreover, improved depth-of-response and increased rates of MRD-negativity have also been observed, raising hopes that some CLL patients could discontinue treatment and experience extended treatment-free periods. However, some questions still remain about how the tumor microenvironment influences venetoclax sensitivity, even in CLL.

According to the 2008 International Workshop for CLL response criteria, a patient's disease must show not only a major reduction in circulating tumor burden (blood lymphocytes <4,000/μL), but also a reduction in the size of all affected lymph nodes (with none measuring >15 mm), an elimination of any splenomegaly or hepatomegaly, and a clearance of the bone marrow (normocellular, with <30% lymphocytes and no B lymphoid nodules) to qualify as a complete response. Therefore, the CRs described in studies to-date indicate that, at least in some CLL patients, venetoclax is able to significantly reduce tumor burden not only in the periphery, but also in primary and secondary lymphatic sites. Although these data seem inconsistent with factors in the lymph nodes and bone marrow mediating resistance to venetoclax, it is not clear whether venetoclax is actually killing tumor cells *in situ* (within these compartments) or simply triggering apoptosis of cells that have temporarily migrated away from their protective niches. It is possible that, when compared to the rapid clearance of circulating tumor cells, the extended amount of time required for venetoclax to clear lymph nodes and bone marrow reflects the protective impact of the microenvironment and the kinetics of tumor cell migration into and out of those niches. The kinetics of venetoclax-mediated reductions in lymphadenopathy and the clearance of disease from bone marrow have not been examined exhaustively, and so it is possible that these effects are actually occurring more rapidly than the current schedule of assessments would indicate. It would be interesting to assess the kinetics and localization of apoptosis by real-time live imaging or other approaches that could shed light on the drug's mechanisms of action.

Because BCL-2 plays such an important role in the development and shaping of the immune system it will also be interesting to explore how venetoclax may impact tumor microenvironments. Might potent BCL-2 inhibition lead to reductions or enrichments in tumor-infiltrating immune cells such as dendritic cells, natural killer cells, myeloid derived suppressor cells, and various B- and T-cell populations? If so, what might be the impact of venetoclax on the efficacy of other immune-modulators? Such combination trials have recently been initiated, and so answers will likely be forthcoming.

These are only a few examples of questions that remain and, clearly, much work remains to be done as we continue to explore ways to harness the activity of BCL-2-selective inhibitors for cancer therapy. As clinical data mature, the oncology community will doubtless continue to refine treatment approaches. At the same time, the hope is that ongoing work in the research community will continue to enhance our understanding of resistance and point the way toward improved therapies for people with cancer.

## Author contributions

JL and DC participated in the conception and writing of this review.

### Conflict of interest statement

JL and DC are employees and shareholders of AbbVie, Inc.
